# Mercury and health care

**DOI:** 10.4103/0019-5278.72240

**Published:** 2010-08

**Authors:** Neeti Rustagi, Ritesh Singh

**Affiliations:** Department of Community Medicine, Maulana Azad Medical College, New Delhi, India

**Keywords:** Mercury, mercury toxicity, occupational hazard

## Abstract

Mercury is toxic heavy metal. It has many characteristic features. Health care organizations have used mercury in many forms since time immemorial. The main uses of mercury are in dental amalgam, sphygmomanometers, and thermometers. The mercury once released into the environment can remain for a longer period. Both acute and chronic poisoning can be caused by it. Half of the mercury found in the atmosphere is human generated and health care contributes the substantial part to it. The world has awakened to the harmful effects of mercury. The World Health Organization and United Nations Environmental Programme (UNEP) have issued guidelines for the countries’ health care sector to become mercury free. UNEP has formed mercury partnerships between governments and other stakeholders as one approach to reducing risks to human health and the environment from the release of mercury and its compounds to the environment. Many hospitals are mercury free now.

## INTRODUCTION

Mercury (Hg) is a heavy silvery-white metal that is found in liquid state at room temperature. It is also known as quicksilver or hydrargyrum. Mercury with atomic number 80 has many characteristic features. It is the only metal that is liquid at standard conditions for temperature and pressure.[1] With a melting point of −38.83°C and boiling point of 356.73°C, mercury has one of the narrowest ranges of its liquid state of any metal. As compared with other metals, it is a poor conductor of heat, but a fair conductor of electricity.[2] Mercury readily vaporizes and may stay in the atmosphere for up to a year. When released into the air, it is transported and deposited globally. Mercury ultimately accumulates at the bottom of water bodies, where it is transformed into its more toxic organic form, methyl mercury, which accumulates in fish tissue. Elemental mercury was known to the ancient Greeks, Romans, Chinese, and Hindus. Each civilization had its own legends about mercury, and it was used as everything from a medicine to a talisman.

## SOURCE AND USES OF MERCURY

Mercury is an extremely rare element in the Earth’s crust. It occurs in deposits throughout the world mostly as cinnabar (Mercuric Sulfide). In 2007, China was the top producer of mercury with almost two-thirds global share followed by Kyrgyzstan.[3] Mercury as a metal is used for extraction of gold and silver, as a catalyst for chlor-alkali production, in manometers for measuring and controlling pressure, in thermometers, in electrical and electronic switches, in fluorescent lamps, and in dental amalgam fillings. Chemical compounds of mercury have found uses in batteries, in biocides in paper industry, in paints, and on seed grain, as antiseptics in pharmaceuticals, as reagents in laboratories, and as catalysts.

Some important mercury salts and their usage are as follows: Mercury (I) chloride (used in medicine, acousto-optical filters and in electrochemistry); Mercury (II) chloride (a very corrosive, easily sublimating, and poisonous substance); Mercury fulminate (a detonator widely used in explosives); Mercury (II) oxide (main oxide of mercury); Mercury (II) sulfide (found naturally as the ore cinnabar or vermilion, a high-grade paint pigment); Mercury (II) selenide, Mercury (II) telluride, Mercury cadmium telluride, and Mercury zinc telluride (semiconductors and infrared detector materials). Organic mercury compounds are also important. Methyl-mercury is a dangerous compound that is widely found as a pollutant in water bodies and streams.[4]

## MERCURY TOXICITY

Mercury gets released into the environment either by volcanic eruptions or by human activities. Volcanic eruptions can increase the atmospheric source by 4–6 times.[5] Natural sources, such as volcanoes, are responsible for approximately half of the atmospheric mercury emissions. The human-generated half can be divided into the following estimated percentages[6] (global human-caused-mercury emissions estimates in 2000):

- 65% from stationary combustion, such as coal-fired power plants.
- 11% from gold production.
- 6.8% from nonferrous metal production, typically smelters.
- 6.4% from cement production.
- 3.0% from waste disposal, including municipal and hazardous waste, crematoria, and sewage sludge incineration.
- 3.0% from caustic soda production.
- 1.4% from pig iron and steel production.
- 1.1% from mercury production, mainly for batteries.
- 2.0% from other sources.

Mercury also enters into the environment through the disposal (eg, land filling, incineration) of certain products. Products containing mercury include the following: auto parts, batteries, fluorescent bulbs, medical products, thermometers, and thermostats.

Mercury is highly toxic, especially when metabolized into methyl mercury. It may be fatal if inhaled and harmful if absorbed through the skin. Around 80% of the inhaled mercury vapor is absorbed in the blood through the lungs. It may cause harmful effects to the nervous, digestive, respiratory, and immune systems, and kidneys and lungs. Adverse health effects from mercury exposure can be tremors, impaired vision and hearing, paralysis, insomnia, emotional instability, developmental deficits during fetal development, and attention deficit and developmental delays during childhood. Mercury and most of its compounds are extremely toxic and are generally handled with care; in cases of spills involving mercury (such as from certain thermometers or sphygmomanometers), specific cleaning procedures are used to avoid toxic exposure.[7] Mercury can cause both chronic and acute poisoning.

The most common potential mode of occupational exposure to mercury is via inhalation of metallic liquid mercury vapors. Since mercury vapor is odorless and colorless, people can breathe mercury vapor unknowingly. For liquid metallic mercury, inhalation is the route of exposure that poses the greatest health risk.

Studies have shown effects, such as tremors, impaired cognitive skills, and sleep disturbance in workers with chronic exposure to mercury vapor even at low concentrations in the range of 0.7–42 *μ*g/L.[8] A study has shown that acute exposure to calculated elemental mercury levels of 1.1–44 mg/m^3^ resulted in chest pain, dyspnea, cough, hemoptysis, impairment of pulmonary function, and evidence of interstitial pneumonitis.[9] The central nervous system is the critical organ for mercury vapor exposure. Subacute exposure has given rise to psychotic reactions characterized by delirium, hallucinations, and suicidal tendency. Occupational exposure has resulted in broad-ranging functional disturbance, including erethism, irritability, excitability, excessive shyness, and insomnia. With continuing exposure, a fine tremor develops and may escalate to violent muscular spasms. Tremor initially involves the hands and later spreads to the eyelids, lips, and tongue. Long-term, low-level exposure has been associated with more subtle symptoms of erethism, including fatigue, irritability, loss of memory, vivid dreams, and depression.[10]

One of the worst industrial disasters in history was caused by the dumping of mercury compounds into Minamata Bay, Japan. The Chisso Corporation, a fertilizer and later petrochemical company, was found responsible for polluting the bay from 1932 to 1968. It is estimated that over 3000 people suffered various deformities, severe mercury poisoning symptoms, or death from what became known as Minamata disease.[11]

## CONTRIBUTION FROM THE HEALTH CARE SECTOR

Health care facilities are one of the main sources of mercury release into the atmosphere because of emissions from the incineration of medical waste. In some places, it is as much as fourth-largest source of mercury in the environment. In the USA, according to the US Environmental Protection Agency (EPA) in a 1997 report, medical waste incinerators were responsible for as much as 10% of all mercury air releases. The water bodies get polluted with the mercury used in the health care sector. Environment Canada estimates that more than one-third of the mercury load in sewage systems is due to dental practice. Dental amalgam is the most commonly used dental filling material. It is a mixture of mercury and a metal alloy. The normal composition is 45%–55% mercury, approximately 30% silver, and the remaining is other metals, such as copper, tin, and zinc. In 1991, the World Health Organization (WHO) confirmed that mercury contained in dental amalgam is the greatest source of mercury vapor in nonindustrialized settings, exposing the concerned population to mercury levels significantly exceeding those set for food and for air.[12] A variety of studies demonstrate that mercury containing health care equipment will invariably break. Small spills of elemental mercury on a smooth, nonporous surface can be safely and easily cleaned up with proper techniques. However, beads of mercury can settle into cracks or cling to porous materials, such as carpet, fabric, or wood, making the mercury extremely difficult to remove. Spilled mercury can also be tracked on footwear. Inadequate cleaning and disposal may expose already compromised patients and health care staff to potentially dangerous exposures.

According to a report submitted to the OSPAR Commission (Convention for the Protection of the Marine Environment of the North-East Atlantic), in the United Kingdom, annually 7.41 t of mercury from dental amalgam are discharged to the sewer, atmosphere, or land, with another 11.5 t sent for recycling or disposed with the clinical waste stream.[13] Together, mercury contained in dental amalgam and in laboratory and medical devices, accounts for about 53% of the total mercury emissions.

An average-sized hospital in India releases around 3 kg of elemental mercury in the environment in a year. With very conservative estimates, a city, such as Delhi, would be releasing around 51kg of mercury each year through dental practices alone.[14]

## ALTERNATIVES TO MERCURY

For many years it was thought that mercury is indispensable. Although there are instruments that are alternative to mercury containing equipments, their use was never widespread. Both mercury and aneroid sphygmomanometers have been in use for many years. The thinking that aneroid sphygmomanometer does not give accurate reading has no base. Of all mercury instruments used in health care, the largest amount of mercury is used in mercury sphygmomanometers (80–100 g/unit), and their widespread use, collectively make them one of the largest mercury reservoirs in the health care setting. By choosing a mercury-free alternative a health care institution can make a tremendous impact in reducing the potential for mercury exposure to patients, staff, and the environment. Aneroid sphygmomanometers provide accurate pressure measurements when a proper maintenance protocol is followed.[15]

## MERCURY-FREE WORK ENVIRONMENT—SUCCESS STORIES

The Governing Council of United Nations Environment Programme (UNEP), in 2003, concluded that there is sufficient evidence of significant global adverse impacts from mercury to warrant further international action to reduce the risks to humans and wildlife from the release of mercury to the environment. The WHO issued a policy paper in 2005 calling for short-, medium-, and long-term measures to substitute mercury-based medical devices with safer alternatives. The World Medical Association passed a resolution in 2008 calling for the substitution of mercury-based medical devices with safer alternatives.

Due to the health effects of mercury exposure, industrial and commercial uses are regulated in many countries. The US Clean Air Act passed in 1990, put mercury on a list of toxic pollutants that need to be controlled to the greatest possible extent. Thus, industries that release high concentrations of mercury into the environment agreed to install maximum achievable control technologies (MACT). Environmental releases and disposal of mercury are regulated in the USA primarily by the US EPA. The WHO, Occupational and Safety Health Administration (OSHA), and National Institute of Safety and Health (NIOSH), all treat mercury as an occupational hazard, and have established specific occupational exposure limits.

The European Union (EU) has banned mercury thermometers for home and health care use beginning in 2008. The EU is considering a similar ban on sphygmomanometers. Argentina’s Minister of Health issued a resolution in February 2009, mandating that all hospitals purchase mercury-free thermometers and sphygmomanometers, and establishing a commission to address mercury in dental practices and to move the country toward a broader mercury phaseout. The Philippines Department of Health issued an administrative order in 2008 calling for the phaseout of mercury-based medical devices across the country by 2010. The Ministry of Health of Uruguay announced in 2009 that it would phaseout the use of mercury thermometers in health care. Taiwan has banned mercury thermometers. Over the past decade, the US health care sector has virtually phased out mercury-based medical devices. It is nearly impossible to purchase a mercury thermometer in the USA today. Sweden, the Netherlands, and Denmark have successfully phased out all mercury-based medical devices, including sphygmomanometers. Since the 1980s, Cuba has implemented a national policy of replacing its mercury sphygmomanometers with aneroid devices.

Many cities in the world are moving toward mercury-free health centers. Growing numbers of hospitals in the developing countries are moving toward mercury-free health care. In Argentina, more than 70 hospitals have replaced or are on the path to replacing mercury-free thermometers and blood pressure devices. In Sao Paulo, Brazil, more than 100 hospitals have eliminated mercury-based thermometers and sphygmomanometers. In the Philippines, more than 50 hospitals are moving toward mercury-free health care. In Mexico, more than 10 hospitals are in the process of replacing, or have already replaced, mercury-based medical devices. Two of the former leading US-based mercury blood pressure device manufacturers, Welch Allyn and Trimline Medical, have ended their production of mercury blood pressure devices.

There are many hospitals in India that are mercury free. The Department of Health and Family Welfare, Government of NCT, Delhi, drafted and circulated a written policy to all the government hospitals, which asks the hospitals to curb the use of mercury equipment. All government hospitals in Delhi have stopped purchase of mercury equipment and are in the process of phasing it out. A nonmercury product replaces any broken mercury instrument. Some of the private hospitals in Delhi had started mercury phaseout as early as 2003 when mercury was included in the training programs conducted for medical waste management.

The WHO is following these strategic steps for an eventually mercury-free health care:

**Short term:** Develop and implement plans to reduce the use of mercury equipment and replace it with mercury-free alternatives. It addresses clean-up, storage, and disposal of mercury.

**Medium term:** Increase efforts to reduce the use of unnecessary mercury equipment in hospitals. Hospitals should have an inventory of their use of mercury. This inventory should be categorized into immediately replaceable and gradually replaceable.

**Long term:** Support a ban of mercury-containing devices and promote alternatives. Support countries in developing a national guidance manual for sound management of health care mercury waste. Support countries in the development and implementation of a national plan, policies, and legislation on mercury health care waste. Support the allocation of human and financial resources to ensure procurement of mercury-free alternatives and a sound management of health care waste containing mercury.
